# The overlapping of phenotypes in Wiedemann-Steiner, Kleefstra and Coffin-Siris syndromes: a study of eleven patients

**DOI:** 10.1186/s13052-024-01763-1

**Published:** 2024-09-19

**Authors:** Elisabetta Prada, Camilla Meossi, Denise Piras Marafon, Federico Grilli, Giulietta Scuvera, Paola Giovanna Marchisio, Carlo Virginio Agostoni, Federica Natacci, Donatella Milani

**Affiliations:** 1 Fondazione IRCSS Ca’ Granda Ospedale Maggiore Policlinico, Milano, Italy; 2 Ospedale Sant’Anna, ASST Lariana, Como, Italy; 3 Department of Developmental Neuroscience, IRCCS Stella Maris Foundation, Pisa, Italy; 4 Università degli Studi di Milano, Milano, Italy

**Keywords:** Intellectual disability, Chromatinopathies, *KMT2A* gene, *EHMT1* gene, *ARID1B* gene, Rest energy expenditure, Indirect calorimetry, Epigenetic machinery

## Abstract

**Background:**

Some chromatinopathies may present with common clinical findings (intellectual disability, brain and limb malformation, facial dysmorphism). Furthermore, one of their cardinal shared features is growth dysregulation.We aimed to assess and deepen this resemblance in three specific conditions, namely Wiedemann-Steiner (WDSTS), Kleefstra (KLEFS1) and Coffin-Siris syndrome (CSS1), with a particular focus on possible metabolic roots.

**Methods:**

Eleven patients were enrolled, three with WDSTS, five with KLEFS1 and three with CSS1, referring to Fondazione IRCCS Ca’ Granda Ospedale Maggiore, Milan, Italy. We performed both a physical examination with detailed anthropometric measurements and an evaluation of the patients’ REE (rest energy expenditure) by indirect calorimetry, comparing the results with age- and sex-matched healthy controls.

**Results:**

We observed new clinical features and overlap between these conditions suggesting that different disturbances of epigenetic machinery genes can converge on a common effect, leading to overlapping clinical phenotypes. The REE was not distinguishable between the three conditions and healthy controls.

**Conclusions:**

Epigenetic machinery plays an essential role both in growth regulation and in neurodevelopment; we recommend evaluating skeletal [craniovertebral junction abnormalities (CVJ) polydactyly], otolaryngological [obstructive sleep apnea syndrome (OSAs), recurrent otitis media], dental [tooth agenesis, talon cusps], and central nervous system (CNS) [olfactory bulbs and cerebellum anomalies] features. These features could be included in monitoring guidelines. Further studies are needed to deepen the knowledge about energy metabolism.

**Supplementary Information:**

The online version contains supplementary material available at 10.1186/s13052-024-01763-1.

## Background

Wiedemann-Steiner syndrome (WDSTS, OMIM #605130), Kleefstra syndrome type 1 (KLEFS1, OMIM #610253) and Coffin-Siris syndrome type 1 (CSS1, OMIM #135900) are ultra-rare genetic conditions associated with mutations in epigenetic regulating genes [1].

While these syndromes have been individually studied, there is a relative lack of comparative studies that analyze them together. This comparative approach enhances our understanding of the underlying pathways and improves diagnostic accuracy and therapeutic strategies for these chromatinopathies.WDSTS is mainly characterized by prenatal and postnatal growth delay, from mild to moderate intellectual disability (ID), localized (cubiti, back, limbs) hypertrichosis, facial dysmorphisms (thick eyebrows, long eyelashes, down-slanting palpebral fissures, hypertelorism, wide nasal bridge and thin lips) and possible cardiac, renal and ocular defects. This condition has been linked to heterozygous mutations in *KMT2A* gene (OMIM *159555) [2].

A typical facies (synophrys/arched eyebrows, up-slanting palpebral fissures, hypertelorism, bulbous nasal tip, midface retrusion, protruding tongue, thick mouth and everted lower lip), microbrachycephaly, hypotonia and speech delay, growth retardation, cardiac defects and moderate to severe ID are typical of KLEFS1 [3], and the diagnosis is established in probands with heterozygous deletions on chromosome 9q34.3 including at least part of *EHMT1* gene (50% of cases) or intragenic *EHMT1* pathogenic variants (50%) [4].

CSS1 patients share the following features: a coarse face, aplasia/hypoplasia of the distal phalanx of the fifth finger and/or absence of the nail, ID of variable degree, growth delay, hypertrichosis, sparse scalp hair, possible malformations of cardiac, genitourinary, gastrointestinal and central nervous systems [5]. Heterozygous mutations in *ARID1B* gene (OMIM *614556) are associated with CSS1.

From a functional point of view, the proteins encoded by the aforementioned genes belong to four different classes of enzymes, each of which participates in the regulation of the chromatin structure and in trascriptional activity [1]. Sebastian et al. suggest that the dysregulation of epigenetic mechanisms can account for several pathologies and contribute to metabolism-associated diseases [6]. The somatic growth is a dynamic process strongly correlated to the energy metabolism. The total energy expenditure (TEE) has three main components: thermogenesis, physical activity and basal metabolic rate (BMR). The latter is the minimal amount of the energy necessary for the preservation of the human homeostasis and amounts to about 40–70% of the TEE. Different factors may affect the BMR, first of all somatic growth; other ones are body surface, body composition, age, sex, ethnicity, body temperature, diet and hormone status. In the clinical practice, the BMR is usually interchangeable with the rest energy expenditure (REE), which is about 10% higher than the BMR and could be measured by indirect calorimetry [7].

In an interesting way, all the aforementioned conditions are mainly characterized by growth delay, albeit with some differences: in all cases a short stature may be typical; however, KLEFS1 individuals commonly become overweight during adolescence, while weight is low or normal in WDSTS and CSS1 [8]. Based on these assumptions, in this paper we describe the clinical features of a group of WDSTS, KLEFS1, and CSS1 patients looking for new and/or overlapping peculiar findings, and assessing their growth pattern to deepen the knowledge of the possible etiopathogenetic determinants. To this purpose, for the first time we investigate the energy metabolism of these syndromes by the evaluation of both anthropometric measurements and REE assessment.

Overall, combining anthropometric measurements with indirect calorimetry provides a comprehensive evaluation of an individual’s energy metabolism and growth status, which is crucial for diagnosing and managing genetic syndromes with metabolic implications.

## Methods

We enrolled pediatric subjects with a clinical and molecular diagnosis of WDSTS, KLEFS1, and CSS1 referred to the outpatient genetic clinic of Fondazione IRCCS Ca’ Granda Ospedale Maggiore, Milan. Genetic analyses, familial, perinatal and childhood history, developmental and cognitive assessment, any other clinical and diagnostic evaluation were collected (Tables 1 and 1S ). A thorough physical examination was then performed with anthropometric measurements and evaluation of REE by indirect calorimetry (Table 2 ). The anthropometric measurements included weight (W), height (H), body mass index (BMI), occipitofrontal (OFC), arm and abdominal circumferences, and bicipital, tricipital, subscapular and suprailiac folds. The indirect calorimetry was performed in the morning, following an overnight fast, using Sensor-Medics Vmax SPECTRA, Milan, Italy. During the exam, each individual had to breathe under a canopy mask for 20–30 min while remaining awake and quiet. The REE values of all patients were compared with the REE of age- and sex-matched healthy controls.

**Table 1:** Comparison of key clinical features between study patients and literature reports

Feature	WDSTS (Study, 3 patient)	WDSTS (Literature)	KLEFS1 (Study, 5 patient)	KLEFS1 (Literature)	CSS1 (Study, 3 patient)	CSS1 (Literature)
**Genetic Mutations**	c.3294G > A, c.3461G > A, c.6873delG in *KMT2A*	> 90% *KMT2A* mutations	Microdeletions (4 patients) and missense mutations (1 patient) in *EHMT1*	80–85% deletions, 20–25% mutations in *EHMT1*	c.5049del, c.3826G > T, c.6164G > A in *ARID1B*	68–83% *ARID1B* mutations
**Facial Dysmorphisms**	Present in all patients	> 80% prevalence	Present in all patients	95% prevalence	Present in all patients	> 90% prevalence
**Hypertrichosis**	Observed in 2 out of 3 patients	60% prevalence	Not observed	Rare	Present in all patients	60% prevalence
**Developmental Delay**	Present in all patients	> 90% prevalence	Present in all patients	> 90% prevalence	Present in all patients	> 90% prevalence
**CNS Anomalies**	Observed in 1 patient	~ 40% prevalence	CNS malformations in 3 patients	50% prevalence	CNS anomalies in 1 patient	50% prevalence
**Gastrointestinal Issues**	Observed in 1 patient	60% prevalence	Observed in 2 patients	~ 30% prevalence	None reported	30% prevalence
**Genitourinary Anomalies**	None observed	30% prevalence	None observed	Rare	1 patient with anomaly	35% prevalence
**Ocular Issues**	Present in 2 patients	60% prevalence	None reported	Rare	Present in 2 patients	40% prevalence
**Dental Issues**	Observed in 1 patient	~ 40% prevalence	None reported	Rare	Delayed dental eruption in 1 patient	40% prevalence
**Recurrent Infections**	Observed in 1 patient	~ 40% prevalence	Recurrent infections in 2 patients	~ 30% prevalence	Frequent infections in 2 patients	60% prevalence

Abbreviations: CNS = Central Nervous System

**Table 2:** Anthropometric measurements and energy expenditures in patients with WDSTS, KLEFS1 and CSS1

	WDSTS (*N* = 3)	KLEFS1 (*N* = 5)	CSS1 (*N* = 3)			
N	Median (Q1;Q3)	N	Median (Q1;Q3)	N	Median (Q1;Q3)	
Weight (DS)	3	-0,59 (Q1 -1,72; 1,31)	5	0,90 (0,76; 1,07)	3	0,06 (-0,67; 0,35)
Height (DS)	3	-0,89 (-1,26; -0,83)	5	-0,75 (-0,77; 0,63)	3	-0,72 (-0,77; -0,14)
OFC (DS)	3	-2,41 (-2,46; -0,07)	5	-1,44 (-1,81; -0,84)	3	1,42 (1,07; 2,10)
BMI (DS)	3	-0,13 (-1,37; 2,14)	5	1,47 (1,18; 2,07)	3	0,95 (-0,28; 0,99)
Arm circumference (cm)	3	18,2 (16,7; 24,5)	3	19,8 (16,2; 28,0)	3	18,8 (16,5; 19,7)
Abdominal circumference (cm)	3	62,0 (Q1 52,3; 82,0)	3	57,0 (48,5; 81,5)	3	55,5 (48,5; 60,5)
Bicipital fold (mm)	3	12 (5; 18)	3	14 (13; 15)	3	10 (5; 5)
Tricipital fold (mm)	3	12 (8; 28)	3	20 (20; 22)	3	16 (15; 19)
Subscapolar fold (mm)	0	0	2	14,5 (12,0; 17,0)	1	3 (3; 3)
Suprailiac fold (mm)	2	15 (5; 25)	3	23 (20; 25)	2	11,5 (8,0; 15,0)
REE (Kcal/die)	3	698 (611; 1348)	5	740 (495; 1394)	3	892 (846; 910)

Abbreviations: OFC = Occipital-Frontal Circumference; BMI = Body Mass Index; REE = Rest Energy Expenditure; DS = Deviation Standard; cm = centimetres; mm = millimetres

The statistical analysis was evaluated using STATA 15.1 software. We calculated the frequencies (number and percentile) for the qualitative variables and the median, the first (Q1) and the third (Q3) quartiles for the quantitative ones. We used the Mann-Whitney test to compare cases and controls. A *p*-value < 0,05 was considered statistically significant.

## Results

We enrolled 11 patients, three with WDSTS, five with KLEFS1 and three with CSS1. Six patients were males (55%) and five were females (45%). The group’s median age was 7.1 years (Q3 3.8; Q5 8.2). All patients had a confirmed molecular diagnosis: mutations in the *KMT2A* gene were found in all WDSTS patients (Tables 1 and 1S); among KLEFS1, four patients had a chromosomal microdeletion partially or fully involving the *EHMT1* gene (80%), while one had a point mutation in *EHMT1* (20%) (Tables 1 and 1S ); all patients with CSS1 had mutations in *ARID1B* (Tables 1 and 1S ).

**Fig. 1:**
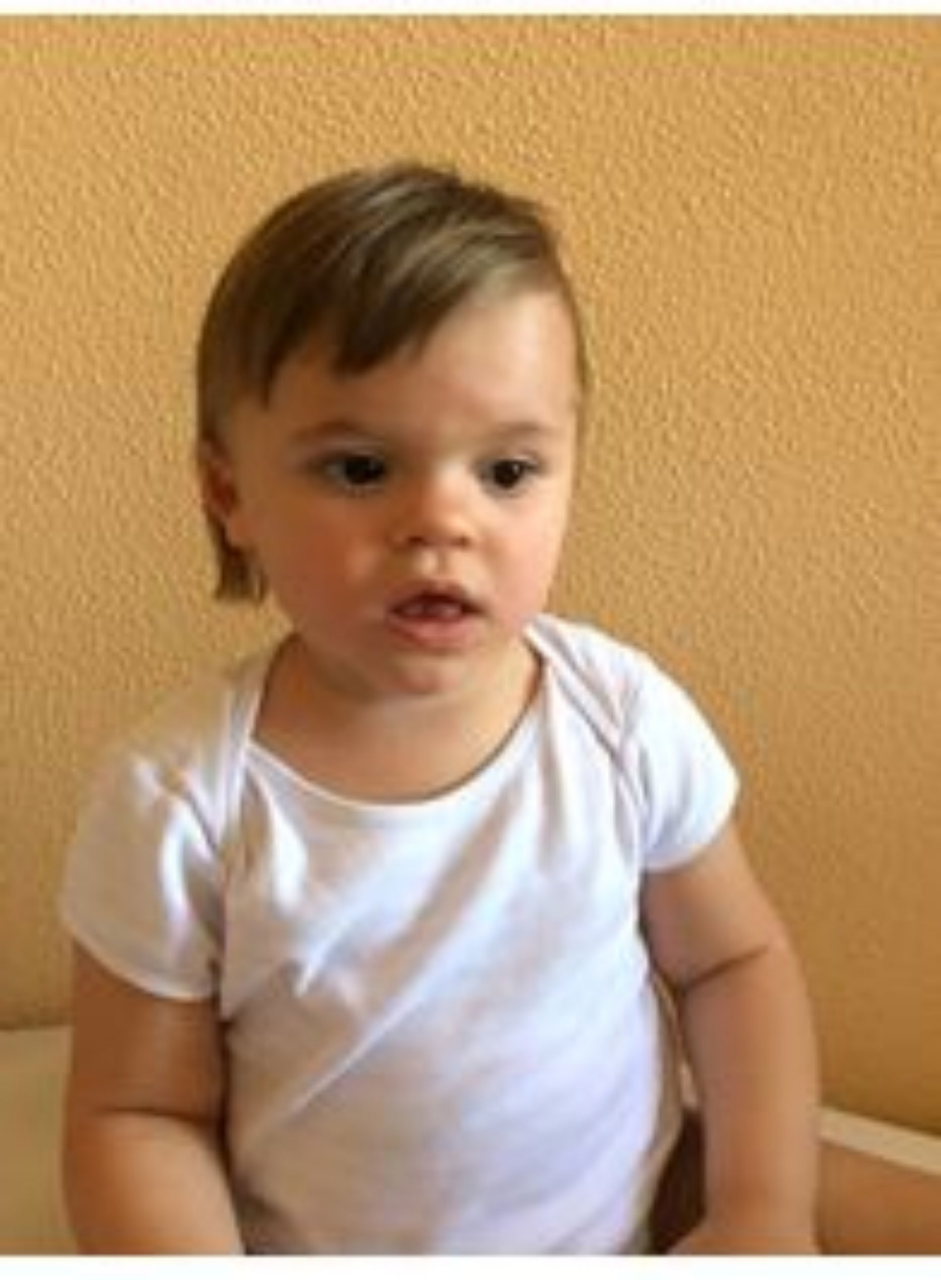
Distinctive facial features in KLEFS1 patient (widely spaced eyes, synophrys, midface retrusion, protruding tongue, eversion of the vermilion of the lower lip, and prognathism of chin)

**Fig. 2:**
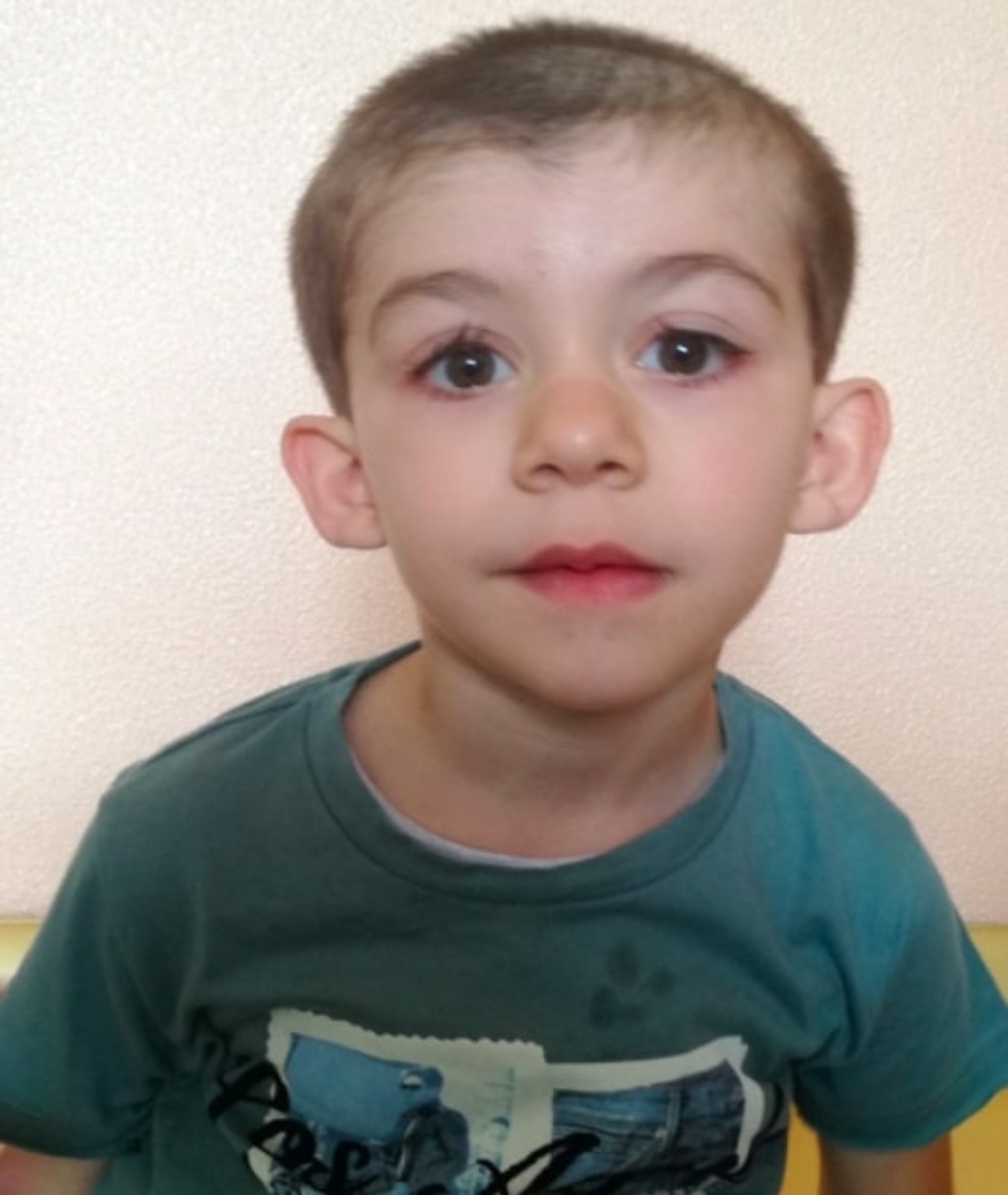
Distinctive facial features in CSS patient (sparse scalp hair, long eyelashes, bulbous nasal tip, anteverted and posteriorly rotated ears, thick and everted lower lip)

Hypertrichosis and neurodevelopmental disorders were evident in all WDSTS patients (Tables 1 and 1S ). Orthopaedic and otolaryngologic involvements were reported in two cases, while ocular, dental, gastrointestinal, and renal defects were found in only one subject. As expected, distinctive facial features (Fig. 1; Tables 1 and 1S ) and neurodevelopmental disorders were common in KLEFS1 group, associated with other clinical issues, especially involving the CNS and musculoskeletal system (four out of five patients). In the CSS1 sample, the cardinal features were present in all individuals (Fig. 2,), who also showed mild CNS malformations (hypolastic, short and dysplastic corpus callosum and small cerebellar vermis), ocular defects, and recurrent infections.

About growth and metabolism, WDSTS patients had a mildly delayed growth, with only microcephaly being significant; KLEFS1 patients presented normal growth but showed higher than normal abdominal fat; CSS1 individuals showed normal anthropometric parameters (Table 2). The indirect calorimetry gave reliable results in all patients with the exception of two cases in which the examination was interrupted due to sleep in one patient (patient 4), and the impossibility to maintain silence in the other one (patient 5).

**Fig. 3:**
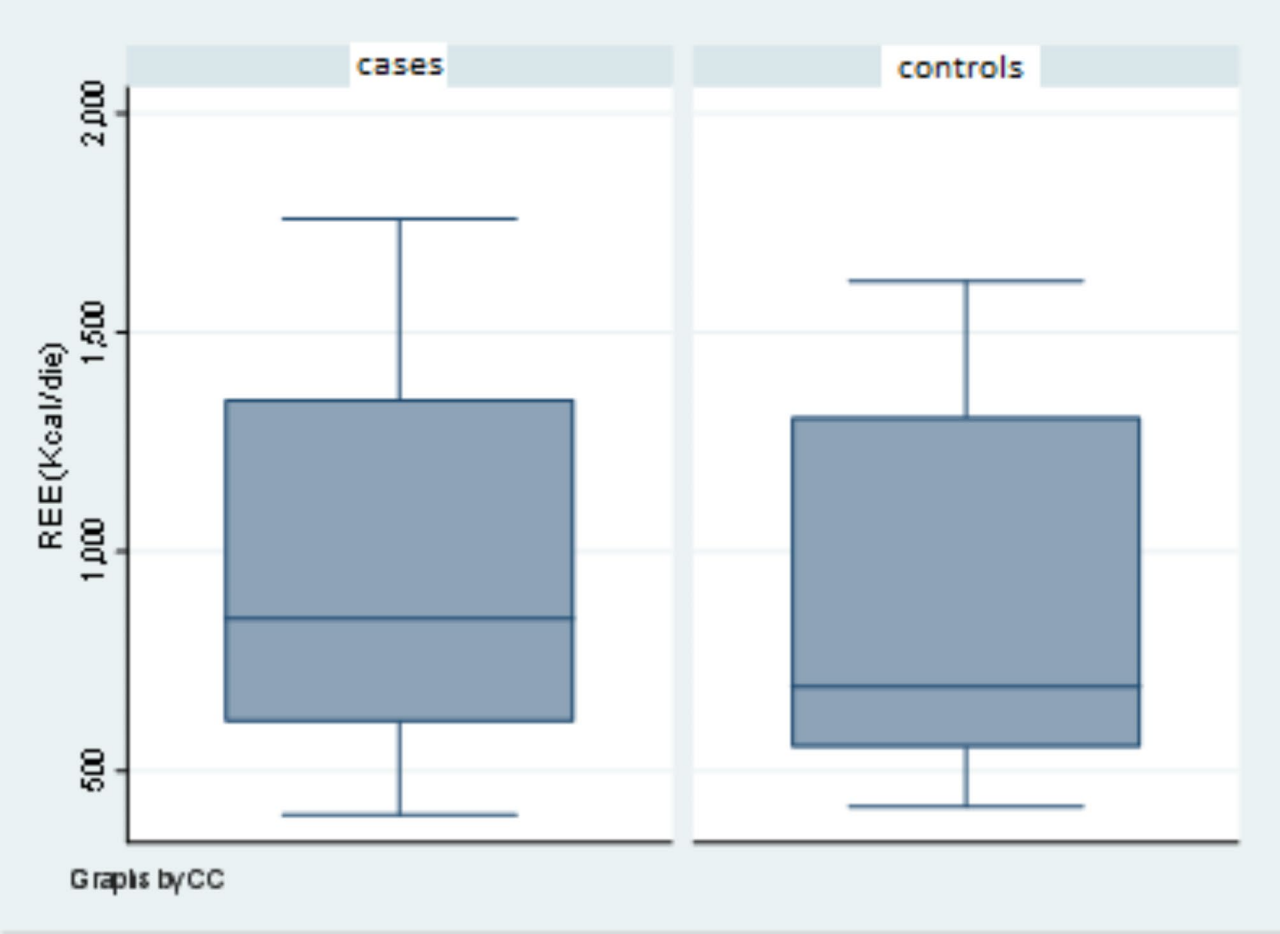
Comparison between the REE of the 11 patients and the 11 age- and sex-matched controls. No statistically significant difference was observed between cases and controls (*p*-value = 0.69)

No statistically significant difference was observed between patients and controls. (*p*-value = 0.69) (Fig. 3).

## Discussion

Different disorders of epigenetic machinery often show common clinical findings; one of the shared cardinal features is undoubtedly growth dysregulation. Based on these assumptions, we compared patients with three Cromathinopaties, searching for overlapping features and investigating metabolism and growth-related issues.

The main diagnostic clues in WDSTS and CSS1 patients are minor anomalies; localized or diffuse hypertrichosis for both and a tiny hypoplasia of the fifth fingernail along with mild specific dysmorphisms such as bulbous nasal tip, thick and everted lower lip for CSS1 [2, 5, 8, 9]. The low clinical burden of these pathognomonic signs carries a risk of an underestimation of cases; when considering KLEFS1 group, we observed instead that the age at diagnosis was lower compared to the other syndromes. A possible explanation could be that most KLEFS1 cases are detected by array-Comparative Genomic Hybridization (a-CGH), which is still often the first-tier genetic test in patients with psychomotor delay. Furthermore we propose that the earlier age at diagnosis observed in KLEFS1 patients could be due to their more pronounced phenotype and the early onset of clear clinical manifestations. This explanation is supported by similar findings in another epigenetic machinery disorder, specifically Sotos Syndrome (SS type 1, OMIM #117550; type 2, OMIM #614753) [14]. In SS, as in KLEFS1, the distinct and early-developing features lead to earlier recognition and diagnosis.

Analyzing the data to highlight overlaps or other signs useful for diagnosis, we noticed a high percentage of WDSTS patients with bone abnormalities, mainly represented by a cervical fusion Klippel Feil-like (2/3; 67%). CVJ and cervical vertebral abnormalities have been recently described in WDSTS patients [9], and are reported in another epigenetic machinery disorder (Rubinstein-Taybi syndrome, RSTS type 1, OMIM **#** 180849; type 2, OMIM **#** 613684) [10]. A further skeletal feature previously outlined in RSTS is preaxial polydactyly. Yu et al. reported polydactyly and syndactyly in nine out of 17 patients with RSTS (37%), with the postaxial polydactyly being more frequent (80%) than the preaxial (20%) in contrast with previous studies [11]. One KLEFS1 patient had a right foot postaxial polydactyly, a peculiar finding, even though extremity abnormalities are commonly described in KLEFS1 patients, mostly being broad toes, syndactyly, clinodactyly and brachydactyly [12].

Moreover, we found two WDSTS patients with adenoid hypertrophy and obstructive sleep apnea (OSA). These clinical features are present in other disorders of Epigenetic Machinery such as RSTS [13] and SS [14].

In all these conditions, OSAs, resulting from a combination of craniofacial dysmorphisms (arched and narrow palate, micrognathia in WDSTS and RSTS while prognathism in SS), easy collapsibility of the laryngeal walls or laryngomalacia, obesity and global hypotonia, can become a major issue, such as during anesthetic procedures. These patients are therefore usually assessed by the otolaryngologist and, if necessary, promptly treated [13, 14]. Three KLEFS1 patients had recurrent otitis media, which has never been reported in this syndrome but frequently described in other epigenetic disorders, such as RSTS [13], SS [14] and CSS1 [5]. Recurrent upper airway infections in these conditions might be facilitated by the anatomic craniofacial conformation and by a peculiar immune status. The latter’s contribution is still being evaluated: Saettini et al. observed immunological abnormalities in about half of 97 RSTS patients, ranging from antibody defects to primary immunodeficiency [15].

Dental abnormalities, such as talon cusps and the absence of molar teeth, were found in our cohort and have been previously described in RSTS1, SS1 and CSS1. These dental abnormalities could also be considered non-invasive signs of Epigenetic Machinery-related disorders.

In addition, we found a bilateral olfactory bulb hypoplasia in one of our KLEFS1 patients. Involvement of the olfactory bulbs is typically associated with CHARGE syndrome (OMIM #214800) [16], another epigenetic disorder caused by mutations in *CHD7* gene (OMIM *608892) [17]. Olfactory anomalies have also been reported in nine of 16 patients with RSTS [10] and in one of 11 patients with Weaver syndrome, (WVS; OMIM #277590), caused by germline pathogenic variants in *EZH2.* WVS *is* a Mendelian disorder characterized by striking overgrowth intellectual disability and distinctive facies [17]. Studies performed on knockout mouse models demonstrated that mutations during the early development in *EHMT1*, *CHD7*, and *CREBBP* (associated with RSTS type 1; OMIM *600140) disrupt the proliferation and the migration of the olfactory epithelium resuling in a size reduction of the olfactory bulbs [18]. Furthermore, as in our cohort, brain Magnetic Resonance Image (MRI) scans of RSTS patients show a high rate (73,6% and 57,8%, respectively) of abnormalities of the corpus callosum and some malformations of the posterior cranial fossa (especially small cerebellar vermis), suggesting a widely overlapped spectrum among these disorders [10].

Lastly, in our cohort of KLEFS1 one patient developed a delay in the right branch conduction, stressing the role of periodic instrumental heart exams even if a first cardiologic evaluation resulted normal; while in our sample of CSS1 one patient had gluten-sensitive enteropathy which may not be linked with the disease, also due to its high incidence in the general population.

Regarding growth and metabolism, the REE measured in our cohort did not significantly differ from that of the age- and sex-matched control group. It should be considered, however, that, although growth impairment is quite common in all these conditions, the trend over time is variable: subjects with WDSTS have both prenatal and post-natal growth delay [2]; individuals with KLEFS1 have short stature and usually become overweight or obese after childhood [3]; patients with CSS1 grow at the lower limits for weight and/or height [5]. Over the last few years, the links between epigenetics and cellular metabolism have been the topics of many studies; impaired epigenetic regulatory mechanisms in cellular metabolism can affect the physiological balance, potentially contributing to diseases. These issues have been studied particularly for obesity, cancer and diabetes [7]. There are no comparable studies, however, regarding chromatinopathies or other growth alteration diseases [19, 20]. Conversely, the energy metabolism has been investigated in other genetic disorders, with contrasting results. Kaplan et al. [21] observed that the REE of six subjects with Williams-Beuren syndrome (WBS, OMIM #194050) was higher than in age- and sex-matched controls. Increased REE values were also reported by Leoni et al. [22] in 11 individuals with Costello syndrome (CSTLO, OMIM #218040). In contrast, patients with Prader-Willi syndrome (PWS, OMIM #176270) showed lower values of energy expenditure [23]. Our cohort’s heterogeneity and the control group’s selection might represent a bias in our evaluation [24]. Indeed, while individuals with WBS and CSTLO may have a lipodystrophic appearance [21, 22] subjects with WDSTS, KLEFS1 and CSS1 have a normal fat distribution (Table 2 ). Assessing fat mass, fat-free mass and their ratios could help evaluate the calorimetric findings in patients with various genetic syndromes.

## Conclusions

We collected the clinical features of a cohort of patients with WDSTS, KLEFS1 and CSS1, observing an intriguing and mazy overlap between these and other disorders of the epigenetic machinery. This prompts us to make some suggestions on the development of diagnostic clues. We suggest that skeletal (CVJ abnormalities, polydactyly), otolaryngological (OSAs, recurrent otitis media), dental (tooth agenesis, talon cusps) and CNS (olfactory bulbs and cerebellum anomalies) features should be investigated and valued in the occurrence of a clinical non-specific hypothesis of chromathinopaty. Prospectively, some of these features could also be considered in the monitoring guidelines of these conditions. Furthermore, this is the first study to investigate the energy expenditure in these conditions, underlining the role of an accurate instrumental evaluation for the purposes of individualized follow-up and the prevention of complications [25–27]. The absence of significant differences in REE between patients and controls makes further studies essential to deepen the knowledge on the impact of chronic conditions as regards metabolic functions over time. Including additional clinical parameters, such as fat distribution and other measurements, could be crucial for a more comprehensive understanding of the patient’s condition. Furthermore, variations in REE could emerge with aging, making regular assessments helpful for a better understanding. In conclusion, also due to the limited sample size, further studies are required to more accurately assess the energy metabolism of these ultra-rare conditions.

## Electronic supplementary material

Below is the link to the electronic supplementary material.

Supplementary Material 1

## Data Availability

The data that support the findings of this study are available on request from the corresponding author. The data are not publicly available due to privacy or ethical restrictions.

## Abbreviations

a-CGH: array-Comparative Genomic Hybridization
BMR: basal metabolic rate
BMI: body mass index
CNS: Central Nervous System
CSS1: Coffin-Siris syndrome type 1
CSTLO: Costello syndrome
CVJ: craniovertebral junction
H: height
ID: intellectual disability
KLEFS1: Kleefstra syndrome type 1
MRI: Magnetic Resonance Image
OFC: occipitofrontal circumference
OSAS: obstructive sleep apnea syndrome
PWS: Prader-Willi syndrome
REE: rest energy expenditure
RSTS: Rubinstein-Taybi syndrome
SPECTRA: Sensor-Medics Vmax
SS: Sotos syndrome
TEE: total energy expenditure
WVS: Weaver syndrome
W: weight
WDSTS: Wiedemann-Steiner syndrome
WBS: Williams-Beuren syndrome
