# Supplementary material for: The overlapping of phenotypes in Wiedemann-Steiner, Kleefstra and Coffin-Siris syndromes: a study of eleven patients
Source: Ital J Pediatr. 2024 Sep 19;50:187. doi: 10.1186/s13052-024-01763-1 (PMC11411858; doi:10.1186/s13052-024-01763-1)
Supplement: Supplementary file 1 — Supplementary Material 1 [file 13052_2024_1763_MOESM1_ESM.docx]

| **Patient ID** | **Age (years)** | **Gender** | **Syndrome** | **Genetic Mutation** | **Key Clinical Features** |
| --- | --- | --- | --- | --- | --- |
| 1 | 8.7 | M | WDSTS | c.3294G>A *KMT2A* | Slanted palpebral fissures, long eyelashes, hypertricosis, Hypotonia, intellectual disability, Hyperintensity of the posterior white matter, Sharp-wave discharges, Extranumerary spleen, Cervical vertebral fusion Klippel Feil-like; metacarpal pseudoepiphysis and metatarsal supernumerary epiphysis; brachydactyly, dental caries and absence of the left molar teeth, Adenoid hypertrophy with obstructive apnea; Raynaud phenomenon |
| 2 | 7.1 | F | WDSTS | c.3461G>A *KMT2A* | Synophrys, hypertrichosis, intellectual disability, stereotypies and aggressivity, Craniocervical dysmorphism, Uncertain malformation Klippel Feil-like; fusion of the first ribs; brachydactyly, Astigmatism and hypermetropy. |
| 3 | 8.2 | M | WDSTS | c.6873delG *KMT2A* | Bulbous nasal tip, enlarged columella, long philtrum, micrognathia, hypertrichosis localized on the back and cubiti, ADHD and oppositivity, Renal pelvis dilated, Talon cusps, Adenoid hypertrophy with obstructive apnea |
| 4 | 14 | M | KLEFS1 | c.3202T>A *EHMT,* | Synophrys, hypertelorism, arched upper lip, prognatism, intellectual disability, Mild ventriculomegaly, Stenosis of pulmonary artery, Scoliosis; left foot pronated, Hypermetropy, Dental caries, Recurrent otitis |
| 5 | 2.8 | F | KLEFS1 | Microdeletion *EHMT1* arr[hg19] 9q34.3 (140714395_  140927911)x1 | Broad and depressed nasal root, anteverted nares, arched upper lip, protruding tongue, prognatism; motor and language delay; Interatrial defect; Conduction delay in the right branch; Bilateral coxa vara; hyperlaxity; Corneal leukoma; Dental tartar; Recurrent otitis |
| 6 | 0.9 | F | KLEFS1 | Microdeletion *EHMT1* arr[hg19] 9q34.3 (140493556_  141005513)x1 | Brachycephaly, up-slanting palpebral fissures, protruding tongue; motor and language delay; Asymmetric ventricles |
| 7 | 3.8 | M | KLEFS1 | Microdeletion *EHMT1* arr[hg19] 9q34.3 (140711171_  140757122)x1 | Synophrys, large and protruding tongue; motor and language delay; Hyperactivity and frustration intolerance; Dysmorphic corpus callosum and ventricles; bilateral periventricular hyperintensity (prenatal injury); Hyperlaxity; Recurrent otitis and short lingual frenulum. |
| 8 | 4 | M | KLEFS1 | Microdeletion *EHMT1* arr[hg19] 9q34.3 (140560792_  141008915)x1 | Hypertelorism, epicanthus, broad nasal root, anteverted nares, wide mouth; motor and language delay; thin corpus callosum and brainstem; hypotrophic cerebellar vermis; hypotrophic olfactory bulbs; postaxial polydactyly on the right foot; hypermetropy; small lower incisors and thin enamel |
| 9 | 3.8 | M | CSS1 | c.5049del *ARID1B* | Brachycephaly, bulbous nasal tip, thick and everted lower lip, hypertrichosis on the back; Hypoplasia of the fifth finger and/or absence of the nail; Sparse scalp hair and hypertrichosis; motor and language delay; Dysmorphic corpus callosum and small cerebellar vermis; Monolateral cryptorchidism; Flat feet; Myopia; Recurrent otitis and upper airway infections |
| 10 | 7.3 | F | CSS1 | c.3826G>T *ARID1B* | Bulbous nasal tip, thick and everted lower lip; Hypoplasia of the fifth finger and/or absence of the nail; Sparse scalp hair and hypertrichosis; motor and language delay; Dysmorphic corpus callosum and small cerebellar vermis; prominent arch of atlas; Celiac disease; hypermetropia; Recurrent otitis and upper airway infections; adenoid hypertrophy with obstructive apnea |
| 11 | 7.8 | F | CSS1 | c.6164G>A *ARID1B* | Bulbous nasal tip, thick and everted lower lip; Hypoplasia of the fifth finger and/or absence of the nail; Sparse scalp hair and hypertrichosis; motor and language delay; short corpus callosum and cerebellar tonsils at foramen magnum; Monolateral double collecting system; Myopia and hypermetropia; Delayed dental eruption; Recurrent otitis and upper airway infections |

**Table 1 (supplementary)** Clinical features in patients with WDSTS, KLEFS1, CSS.

**Abbreviations:** M=male; F=female; ADHD=Attention Deficit Hyperactivity Disorder
